# Giant calcified chronic subdural hematoma adherent to the sagittal sinus: A case report and surgical considerations

**DOI:** 10.1097/MD.0000000000046124

**Published:** 2025-11-21

**Authors:** Menghui He, Xiaoqing Jin, Chunming Xian, Zhongsheng Lu, Qiang Zhang, Pei Han

**Affiliations:** a Department of Graduate School, Qinghai University, Xining, China; b Department of Neurosurgery, Qinghai Provincial People’s Hospital, Xining, China.

**Keywords:** calcification, chronic, subdural hematoma, surgical procedures

## Abstract

**Rationale:**

Calcified chronic subdural hematoma (CCSDH) is a rare neurosurgical condition accounting for 0.3% to 2.7% of all chronic subdural hematomas (CSDH). CCSDH typically manifests in the cerebral convexity, particularly the frontal and parietal lobes. This lesion forms primarily through gradual calcification of unabsorbed CSDH over extended periods.

**Patient concerns:**

A 33-year-old male patient, with a history of head trauma 4 years ago, subsequently developed intermittent right frontoparietal headaches, which were relieved by rest.

**Diagnoses:**

The cranial computed tomography scan revealed a mixed-density space-occupying lesion measuring approximately 9.9 cm × 5.7 cm in the right frontoparietal region. It was characterized by a thick and incomplete arc-shaped calcification layer, enclosing a hematoma in various stages of tissue or calcification. Further cranial magnetic resonance imaging examination showed that the lesion was oval-shaped, presenting as a heterogeneous low signal on T1-weighted images and a high signal on T2-weighted images. Histological examination of the excised tissue confirmed the presence of a calcified hematoma. Based on the above imaging and histological findings, a diagnosis of a giant CCSDH was established.

**Interventions and outcomes:**

The patient underwent a craniotomy to remove the lesion. The surgical procedure was smooth, with no complications occurring. Postoperative recovery was stable, and follow-up imaging confirmed near-complete resection of the lesion. During the 14-month follow-up period, the patient remained asymptomatic with no recurrence.

**Lessons:**

This case highlights the rarity and complexity of CCSDH attached to the sagittal sinus, emphasizing the importance of early recognition and surgical intervention. A literature review indicates that while surgical intervention can significantly improve the prognosis of symptomatic patients, the risks and benefits must be carefully weighed for asymptomatic cases.

## 1. Introduction

Chronic calcified subdural hematoma (CCSDH) is a relatively rare and challenging condition to recognize clinically. The widespread occurrence of subdural calcification or ossification in the cerebral cortex region is commonly referred to as “armored brain.”^[1,2]^ This pathological phenomenon was first documented by Von Rokitansky in 1884 during an autopsy, with subsequent continuous reports. The pathogenesis of CCSDH remains incompletely understood. Existing studies suggest possible pathophysiological factors, mainly including trauma, degenerative changes, local circulatory disturbances, and inflammatory responses.^[3]^ Most cases are trauma-related, but the traumatic event may be overlooked due to its distant occurrence or subtle symptoms.^[4]^ Long-standing hematomas (typically over 6 months) can lead to malabsorption, hyaline degeneration of fibrous tissue, and calcium salt deposition, ultimately causing calcification.^[5]^ Calcification may initiate with hyaline degeneration in the inner layer of the hematoma, gradually progressing to calcification and even ossification (the terminal stage of calcification).^[6]^ The most common manifestation of CCSDH is a space-occupying lesion over the cerebral hemisphere. Rarely, it may involve the tentorium cerebelli, the vicinity of the falx cerebri, or other regions, but such cases are uncommon.^[7]^ Multiple case records indicate that the diameter of CCSDH usually ranges from 15 to 65 mm. This article presents a case of a massive CCSDH in the right frontoparietal region, with a maximum extent of approximately 99 mm × 59 mm × 57 mm, which is relatively uncommon in previous reports.

**Figure 1.**
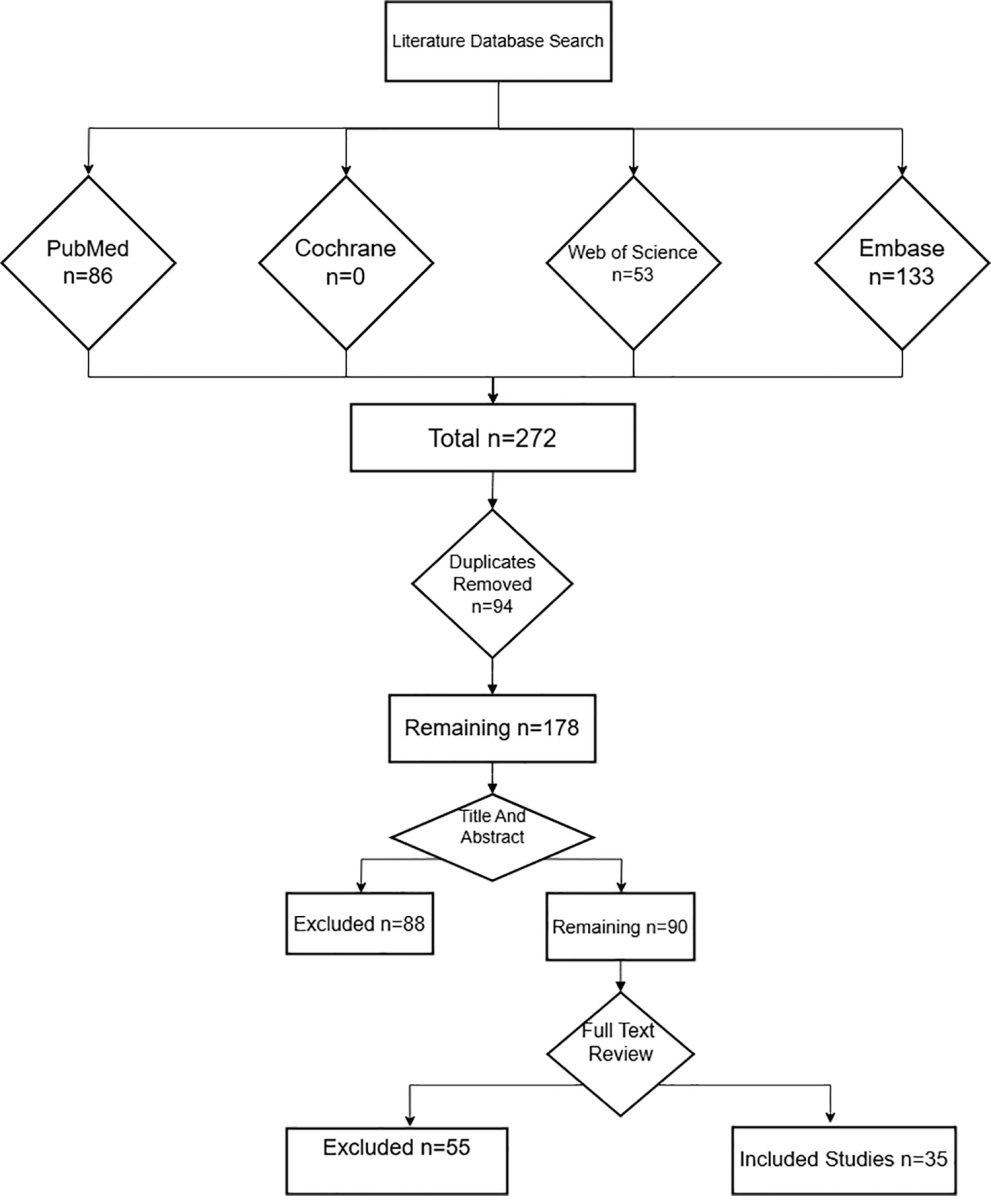
Flow chart of literature screening.

We conducted a systematic search of 4 authoritative databases and collected all CCSDH reports by setting search terms (Fig. 1). A total of 272 reports were retrieved, and after duplicates were removed, 178 remained. A full-text review revealed that only 35 reports with relatively complete data were included. Subsequently, a review of the included literature was performed.

## 2. Case reports

### 2.1. History

**Figure 2.**
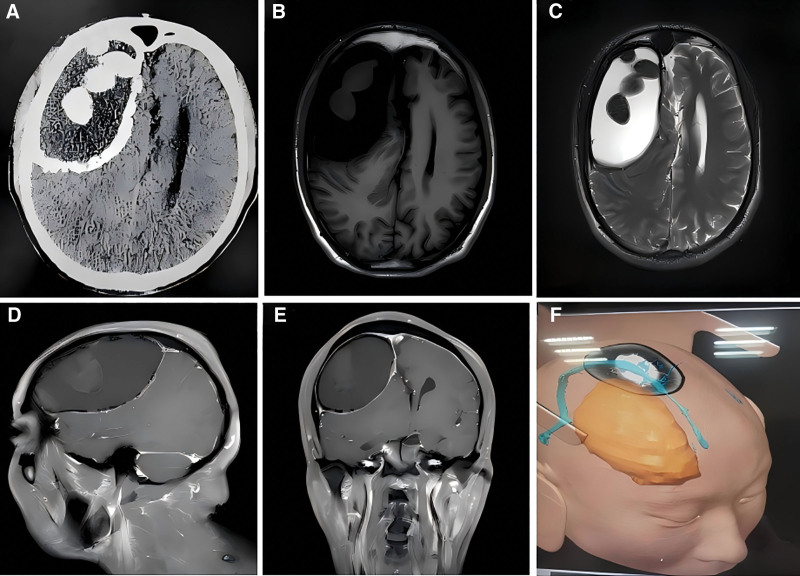
Preoperative cranial CT and MRI examination. (A) Cranial CT scan. (B) Cranial MRI T1-weighted axial image. (C) Cranial MRI T2-weighted axial image. (D) Cranial MRI sagittal image. (E) Cranial MRI coronal image. (F) 3D reconstruction image under neuronavigation. CT = computed tomography, MRI = magnetic resonance imaging.

A 33-year-old male patient had a documented head trauma history 4 years ago, followed by intermittent right frontoparietal distending pain that resolved spontaneously. Neurological examination revealed no significant positive signs. Computed tomography (CT) imaging demonstrated a mixed-density space-occupying lesion in the right frontoparietal region, measuring approximately 9.9 cm × 5.7 cm, surrounded by a thick, incomplete arc-shaped calcified layer (5 mm) encasing hematomas in varying stages of organization or calcification (Fig. 2A). Magnetic resonance imaging (MRI) revealed an oval-shaped lesion with heterogeneous T1 hypointensity (Fig. 2B) and T2 hyperintensity (Fig. 2C). The sagittal (Fig. 2D) and coronal (Fig. 2E) MRI views clearly demonstrate the morphological characteristics of the lesion and its spatial relationship with the adjacent brain tissue. Based on the patient’s various imaging data, a 3D model was constructed under neuronavigation, allowing clear observation of the hematoma adjacent to the sagittal sinus (Fig. 2F).

### 2.2. Surgical procedure

**Figure 3.**
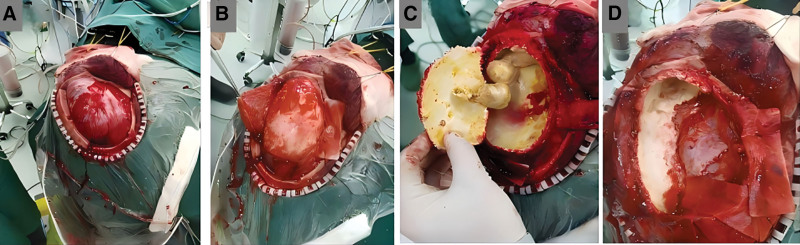
The surgical procedure for CCSDH. (A) Remove the bone flap. (B) Incise the dura mater. (C) Remove the contents. (D) Remove the calcified lesion. CCSDH = chronic calcified subdural hematoma.

**Figure 4.**
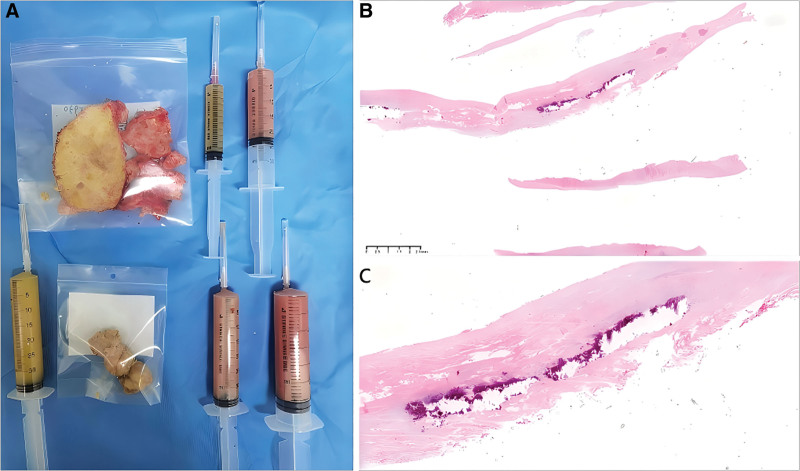
Intraoperative removal of the hematoma and pathological examination. (A) Pathological specimen. (B, C) Pathological examination images.

Preoperatively, neuronavigation was used to mark the hematoma’s location and boundaries on the scalp. The patient was positioned supine with the head tilted left, and a right frontotemporal parietal “U”-shaped craniotomy was performed. After the bone flap was removed, the dura mater with high tension and the calcified tissue beneath it could be palpated (Fig. 3A). Upon dural incision, a smooth, well-demarcated, eggshell-like calcified lesion protruding from the brain surface was observed. The lesion exhibited poor mobility and significant compression of adjacent brain tissue and vasculature (Fig. 3B). The calcified lesion was opened with rongeurs, releasing abundant yellow turbid cystic fluid (Fig. 4A). Near the frontal base, multiple round gray-yellow cystic masses were observed, which, upon incision, revealed yellow oily contents (Fig. 3C). After evacuating cystic contents, the lesion’s visceral layer was found to be densely adherent to the brain and surrounding dura. The basal calcified focus was sequentially removed with rongeurs. Midline calcified tissue displayed intimate adherence to the sagittal sinus (Fig. 3D), with considerable bleeding and removal difficulty. Therefore, a portion of the calcified tissue was left behind, and the dura mater defect was repaired with a biological membrane. After hematoma resection, we performed cranial reconstruction and reduction.

### 2.3. Postoperative course

**Figure 5.**
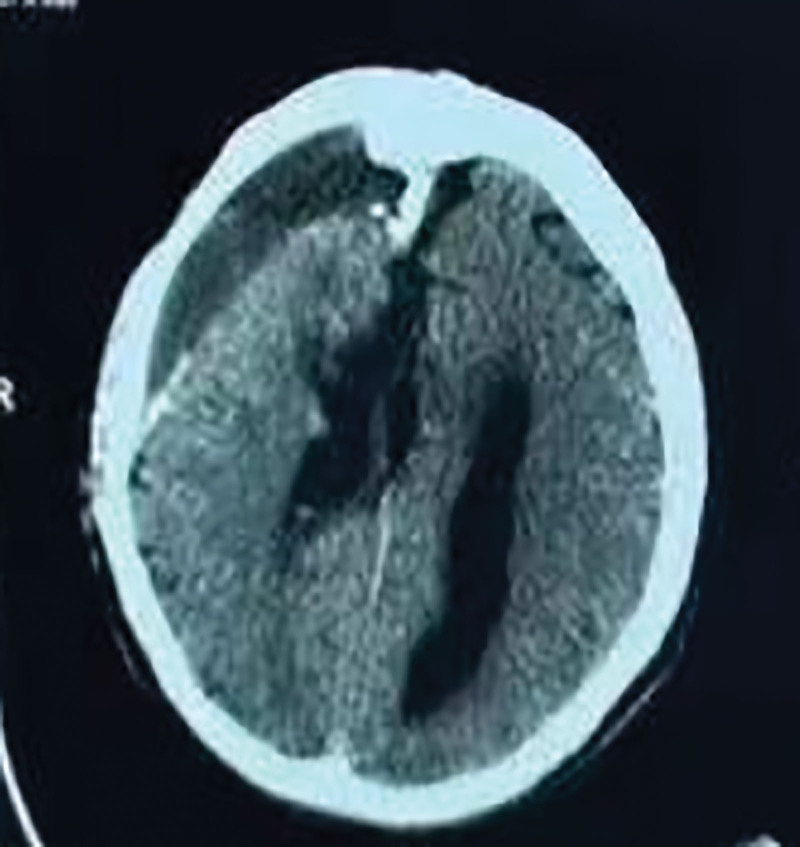
Postoperative follow-up cranial CT. CT = computed tomography.

Postoperatively, the patient was transferred to the intensive care unit and experienced no surgical complications. Histopathological examination of the resected material confirmed a calcified hematoma (Fig. 4B, C). Two weeks later, the patient’s condition improved sufficiently for discharge. A follow-up cranial CT scan 1 month post-surgery (Fig. 5) confirmed near-complete lesion removal. After 14 months of follow-up, the patient remained asymptomatic with no recurrence.

### 2.4. Literature review

Through systematic retrieval and screening, we identified 35 reports with relatively complete information, encompassing 42 patients (including the present case). Table 1 summarizes the baseline characteristics of all included patients. Statistical analysis revealed that CCSDH onset ranges from 3 to 86 years, with a bimodal age distribution and a mean age of 41.88 years (Table 2). Specifically, 14 cases involved minors (3–18 years) and 15 cases elderly individuals (60–90 years). No demographic or genetic susceptibility patterns have been reported to date. Our data indicate a male predominance, with a male-to-female ratio of approximately 3.1:1.1. Regarding medical history, 13 patients had undergone ventriculoperitoneal shunting, 11 had craniocerebral trauma, and 3 had meningitis.^[1,8,9]^ Initial presentations predominantly involved mass effect symptoms, including intracranial hypertension, impaired consciousness, and limb weakness (19 cases, 52.4%). Mild headaches or asymptomatic cases accounted for 10 (23.8%), while epileptic seizures were observed in 10 (23.8%), and atypical symptoms in 3. Of the 42 cases, 12 (28.6%) exhibited bilateral cerebral hemisphere involvement. Approximately 92.9% of CCSDH cases spanned 2 to 3 cerebral lobes, with only 3 cases localized to a single lobe.^[10–12]^ However, apart from our patient, the feature of lesion adhesion to the sagittal sinus was not reported in the remaining 41 cases.

**Table 1:** Literature review of calcified chronic subdural hematoma.

Author (yr)	Sex	Age (yr)	Incentive	Disease period	Location	Symptom	Surgery	Postoperative complications	Outcome	FU (M)	Recurrence
Ludwig (1983)^[1]^	M	34	Meningitis	33 yr	BFP	Gait disturbance, visual impairment	No intervention	–	Stable status	–	–
	F	37	–	27 yr	BFP	HA, nausea	No intervention	–	Stable status	–	–
	F	3	Shunt	3 yr	LFTP	Seizure	Craniotomy	Seizure, right hemiparesis	Stable status	–	–
	F	8	Shunt	7 yr	BTP	Asymptomatic	No intervention	No	Stable status	–	–
	M	18	Shunt	11 yr	LF	Seizure, high intracranial pressure	Craniotomy	Hydrocephalus and shunt	Stable status	–	–
Esayas (2023)^[3]^	M	84	–	8 mo	RFTO	Right LW, HA	Craniotomy	No	Improved	6	No
Ruben (2007)^[8]^	M	67	Meningitis	6 mo	BFT	Bilateral oculomotor nerve palsy	Craniotomy	Hemorrhage	Aggravated	1.5	Yes
Wei-Sheng (2011)^[9]^	M	10	Meningitis	7 yr	LFP	Seizure	Craniotomy	No	Improved	36	No
	F	35	Shunt	30 yr	RFTP	Coma	Craniotomy	No	Improved	12	No
Musharaf (2025)^[10]^	M	73	–	—	LP	Right eye vision loss	Craniotomy	No	Improved	–	No
Huaqiang (2019)^[11]^	M	15	–	15 d	RF	HA, dizziness	Craniotomy	No	Improved	–	–
Shinri (2010)^[12]^	F	32	–	4 yr	LP	HA, impaired consciousness, gait instability	Craniotomy	No	Improved	36	No
Galldiks (2009)^[13]^	M	86	Trauma	3 mo	BFTO	HA, personality change	Burr hole	No	Improved	–	–
Huan (2017)^[14]^	M	61	Trauma	22 yr	LFP	HA	Burr hole, then craniotomy	Intracranial infection	Improved	–	–
Pakrasi (2021)^[15]^	M	75	–	18 mo	RFP	Left hemiparesis, acute unconscious	Craniotomy	Aspiration pneumonia	Died	–	–
Piyaporn (2020)^[16]^	M	83	Trauma	3 mo	RFP	Dysarthria, dyspnea, left LW	Craniotomy	Ventilator-associated pneumonia	Died	–	–
Kyung-Sub (2007)^[17]^	F	47	–	–	RFPO	HA, dizziness	Craniotomy	Contralateral acute subdural hematoma	Improved	2	No
Tatli (2006)^[18]^	M	16	–	–	LFP	HA, seizure, mild right LW	Craniotomy	Contralateral acute subdural hematoma	Improved	24	No
Panagiotis (2008)^[19]^	M	33	Shunt	1 yr	BTO	High intracranial pressure	VP shunt	No	Improved	–	–
Lia (2013)^[20]^	M	73	–	18 mo	LFTO	Right LW	Craniotomy	No	Improved	3	No
Pavol (2020)^[21]^	F	81	–	–	LFTO	HA, confusion and right LW	Craniotomy	No	Improved	7	No
Puneet (2013)^[22]^	F	15	–	6 wk	BF	HA, seizure	Bilateral craniotomy	No	Improved	12	No
Jun (1988)^[23]^	M	25	–	–	LFP	Asymptomatic	Craniotomy	No	Stable status	10	Yes
Matsumura (1984) ^[24]^	M	77	Trauma	37 yr	LFTP	Asymptomatic	No intervention	—	Stable status	–	–
Asifur (2012)^[25]^	M	65	Trauma	20 yr	RFTP	Left LW, confusion	Craniotomy	No	Improved	–	–
Toshihiko (1989)^[26]^	F	59	Trauma	10 yr	RFP	Left hemiplegia, impaired consciousness, seizure	Craniotomy	No	Improved	–	–
Huseyin (2006)^[27]^	M	4	Trauma	2 yr	RPT	HA, head deformities	Craniotomy	No	Improved	–	–
Shuxin (2018)^[28]^	M	60	Trauma	28 yr	LFP	Right LW, HA	Craniotomy	No	Improved	6	No
Sharma (1999)^[29]^	M	10	Shunt	9 yr	BTTO	HA, vomiting, impaired consciousness, left-sided mild hemiplegia	Craniotomy	No	Improved	12	No
	M	9	Shunt	9 yr	RFP	Vomiting, impaired consciousness, left spastic hemiplegia	Craniotomy	No	Improved	6	No
Romel (2008)^[30]^	F	30	Shunt	29 yr	LPO	Seizure, HA, confusion	No intervention	—	Improved	24	No
Alessandra (2020)^[31]^	M	15	Shunt	9 mo	RFPO	HA, vomiting, left LW	Craniotomy	No	Improved	3	No
Ali (2011)^[32]^	M	7	Shunt	9 mo	BFTP	HA, seizure, intellectual decline	No intervention	—	Stable status	–	–
Yamashima (1987)^[33]^	M	75	Trauma	6 mo	LFP	Dementia, gait disturbance	Craniotomy	No	Improved	–	–
Mahmoud (2012)^[34]^	M	12	Shunt	2 yr	BO	Seizure	No intervention	—	Stable status	12	–
Elizabeth (2024)^[35]^	M	49	–	8 mo	LFTO	Seizure, right LW	Craniotomy	No	Improved	3	No
Takeshi (1989)^[36]^	M	62	–	10 yr	LFP	Trigeminal neuralgia, facial tic	Craniotomy	No	Improved	20	No
Hakan (2010)^[37]^	F	17	Shunt	3 yr	BF	Severe HA, vision loss	Craniotomy	No	Improved	36	No
Zhengxi (2010)^[38]^	M	75	–	–	LFP	HA, right LW, dizziness, nausea, vomiting	Craniotomy	No	Improved	12	No
Jun (2022)^[39]^	M	46	Trauma	15 yr	LFTP	Personality change, HA, head swelling	Craniotomy	No	Improved	6	No
Dimogerontas (2006)^[40]^	M	43	Shunt	40 yr	BFOP	HA, vomiting, fever, impaired consciousness	VP shunt	No	Improved	24	No
Our 1	M	33	Trauma	4 yr	RFTP	HA	Craniotomy	No	Improved	14	No

“—” = No relevant data, B = indicates bilateral, F = female, F = frontal, FU = follow-up, HA = headache, L = left, LW = limb weakness, M = male, O = occipital lobe, P = parietal, R = right, T = temporal.

**Table 2:** Statistical table of data results.

	n (%)
Patients	42
Male	31 (73.8%)
Female	11 (26.2%)
Mean/median age (SD, range), yr	41.88/36 (26.82, 3–86)
Etiology	
VP shunt	13 (31.0%)
Trauma	11 (26.2%)
Meningitis	3 (7.1%)
No mentioned	15 (35.7%)
Symptoms	
Headache	21 (50.0%)
Seizure	10 (23.8%)
Limb weakness	14 (33.3%)
Unconscious	10 (23.8%)
Nausea, vomiting	7 (16.7%)
Asymptomatic	3 (7.1%)
Other	8 (19.0%)
Position	
Bilateral cerebral hemispheres	12 (28.6%)
Unilateral cerebral hemisphere ≥ 2 lobes	27 (64.3%)
1 lobe only	3 (7.1%)
Surgery	
Craniotomy	32 (76.2%)
Burr hole	2 (4.8%)
VP shunt	2 (4.8%)
No	7 (16.7%)
Complication	
Pneumonia	2 (4.8%)
Intracranial infection	1 (2.4%)
Contralateral acute subdural hematoma	2 (4.8%)
Other	3 (7.1%)
Outcome	
Improved	30 (71.4%)
Stable status	9 (21.4%)
Aggravated	1 (2.4%)
Died	2 (4.8%)
Recurrence	
Yes	2 (4.8%)
No	22 (52.4%)
No mentioned	18 (42.8%)

SD = standard deviation.

Among the 42 study subjects, 32 underwent craniotomy, 1 patient^[13]^ underwent bilateral burr hole drainage, and 2 patients underwent ventriculoperitoneal shunt surgery, while 7 patients received no surgical intervention. Our patient showed significant improvement in symptoms after undergoing craniotomy, with no complications observed. Additionally, 1 patient^[14]^ developed intracranial infection after 2 burr hole drainage procedures and experienced symptom improvement following subsequent craniotomy. Among the 32 craniotomy patients, 2 died of aspiration pneumonia^[15]^ and ventilator-associated pneumonia,^[16]^ respectively. One patient recovered well after the initial craniotomy, but experienced an increase in the size of the in situ hematoma 6 weeks postoperatively, and was out of danger after a subsequent craniotomy, with no recurrence during the follow-up period. Additionally, 2 patients^[17,18]^ developed acute subdural hematomas on the contralateral side following craniotomy, and their symptoms improved after emergency craniotomy was performed again. The condition of the 7 patients who did not undergo surgical intervention did not show further deterioration. The symptoms of the 2 patients who underwent VP shunt surgery also showed significant improvement.

## 3. Discussion

### 3.1. Epidemiology of CCSDH

The overall incidence of CCSDH is extremely low, constituting only 0.3% to 2.7% of all CSDH cases.^[19]^ In recent years, the number of reported CCSDH cases has declined compared to earlier periods, with ossified chronic subdural hematoma being particularly rare.^[41]^ This trend may be attributed to the widespread use of CT and MRI, enabling early detection and treatment of CSDH during its noncalcified phase, thereby reducing the likelihood of progression to calcification.^[20,21]^ Advancements in early diagnosis and treatment suggest that calcified-phase cases may decrease in the future. Long-term cohort studies or regional epidemiological investigations are required to validate this hypothesis.

### 3.2. Imaging manifestations

CT imaging of CCSDH typically reveals intracystic or cyst wall calcifications within subdural hematomas, appearing as high-density regions that starkly contrast with surrounding brain tissue.^[22]^ The lesion exhibits a well-defined encapsulated structure with mixed density variations. A prominent thick, clearly demarcated, and hemispherically distributed high-density calcified layer surrounds the lesion, serving as a key diagnostic imaging feature. Asymptomatic individuals may display isolated calcified foci without significant mass effect,^[23,24]^ whereas symptomatic patients (e.g., with headache or hemiplegia) often show imaging evidence of brain tissue compression.^[25]^ Large hematomas can induce midline shift and, in severe cases, cerebellar tonsillar herniation. On MRI, the calcified capsule typically appears as low or isointense on T1-weighted images and low signal on T2-weighted images, while the hematoma interior may exhibit mixed signals due to varying hemorrhage stages. MRI effectively delineates hematoma boundaries, extent, and associated brain tissue compression. Imaging-based differential diagnoses include calcified epidural hematoma, meningioma, and calcified arachnoid cyst. CT is the preferred imaging modality for the diagnosis of CCSDH, and when combined with MRI, it can comprehensively assess the degree of calcification, mass effect, and changes in surrounding structures of the hematoma.

### 3.3. Clinical characteristics

In a review of 42 CCSDH cases from the included literature, the average disease duration was 10.3 years. In elderly patients, trauma is the primary etiology, whereas in younger patients,^[26–28]^ shunt surgery is often implicated.^[29–31]^ Due to the initial hematoma’s lack of mass effect, CCSDH typically lacks specific clinical manifestations for an extended period. Additionally, brain atrophy in middle-aged and elderly individuals may provide compensatory space, mitigating hematoma compression effects. CCSDH can induce a range of symptoms, including headache, seizures, neurodevelopmental delays, hemiplegia, and gait abnormalities. These symptoms are primarily attributed to cerebral atrophy and hematoma-related compression. Approximately 50% of patients present with headache as the initial or primary symptom, often persisting for months to years. Patients may experience a decline in consciousness level, with some patients accompanied by cognitive dysfunction, such as memory deficits and dementia.^[32,33]^ About 23.8% of patients develop epileptic symptoms, potentially linked to cerebral cortex irritation or calcification.^[34,35]^ Notably, a subset of patients may exhibit atypical symptoms due to hematoma compression, such as trigeminal neuralgia,^[36]^ oculomotor nerve palsy, and visual disturbances.^[37]^ CCSDH can compress or damage the trigeminal nerve, oculomotor nerve, and visual pathways through mass effect, mechanical irritation, inflammation, or ischemia, leading to corresponding symptoms. With appropriate intervention, patients often achieve partial or complete neurological recovery.

### 3.4. Therapeutic strategies

Currently, there is no unified consensus on the treatment of CCSDH. For patients with mild and stable neurological dysfunction, CCSDH may have ceased progression and is accompanied by brain tissue atrophy. In such cases, surgical intervention has limited impact on improving long-term brain atrophy, as the atrophied brain parenchyma is often difficult to fully expand, and the increased brain volume post-surgery may lead to the formation of subdural fluid collections.^[42]^ However, surgical intervention is necessary when CCSDH exerts continuous compression on brain tissue, exacerbating neurological deficits. Multiple studies indicate that craniotomy or burr hole drainage for hematoma evacuation significantly alleviates symptoms and improves neurological function. There remains considerable controversy regarding the necessity of surgical treatment for asymptomatic CCSDH. For younger patients, the calcified capsule may cause prolonged brain tissue compression, leading to adhesion or cortical damage; thus, prophylactic surgery is advisable to prevent long-term complications. Additionally, the calcified hematoma capsule exhibits significant vascular proliferation, indicating a potential risk of hemorrhage, and surgical treatment is generally considered the preferred intervention. It is noteworthy that postoperative hemorrhage and infection are the most common complications of CCSDH surgery. In the literature review, 2 patients died of pulmonary infection despite successful surgeries, indicating that adequate preoperative preparation and postoperative care are critical factors influencing prognosis.

A review of the literature and clinical data indicates that craniotomy is effective for CCSDH patients with extensive hematoma capsule calcification, organized or compartmentalized contents, complex septations, or failed/recurrent burr hole drainage. By fully exposing the surgical field through craniotomy, the calcified capsule is removed and the hematoma contents are evacuated. This reduces postoperative dead space and promotes brain tissue re-expansion.^[38,39]^ Traditional burr hole drainage is suitable for ordinary CSDH, but CCSDH with calcified capsules is difficult to drain, allowing only partial removal of liquefied components. This fails to relieve the “constraint” effect on brain tissue and has a high recurrence rate. For elderly patients or those intolerant to craniotomy, burr hole drainage can alleviate some neurological symptoms. Juan et al^[9]^ reported a novel therapeutic approach for treating CCSDH – multiple tenting techniques. This technique involves suspending and fixing the calcified inner and outer membranes to the dura mater, allowing the dead space to collapse and close, thereby reducing hematoma recurrence without increasing the risk of new brain injuries.

In addition, VP shunt surgery effectively reduces intracranial pressure by draining cerebrospinal fluid and maintains the dynamic balance of cerebrospinal fluid, thereby alleviating the patient’s symptoms.^[40]^ For patients with mild and stable neurological dysfunction, it is crucial to weigh the risks of long-term compression against the benefits of surgery, and individualized decision-making is essential.

Complete removal of CCSDH presents significant technical challenges. When the hematoma’s calcified capsule is tightly adherent to the dura mater and cerebral cortex, dissection risks dural injury, cerebral contusion, and hemorrhage. Particularly in larger CCSDHs, adhesion to critical vessels like venous sinuses can cause severe intraoperative bleeding. For instance, in our reported case, some calcified tissue was intimately attached to the sagittal sinus, making subtotal resection more feasible. While complete CCSDH removal is technically demanding, optimizing surgical techniques, perioperative care, and individualized strategies can markedly enhance patient outcomes.

## 4. Conclusion

This study presents a rare case of giant CCSDH adherent to the sagittal sinus and a literature review. Results indicate that surgical intervention significantly improves neurological function in CCSDH patients with acute neurological deficits. However, management of asymptomatic CCSDH requires careful consideration, including evaluating patient age, hematoma size, calcification extent, and surgical risks. Future research could integrate these critical factors in surgical decision-making with artificial intelligence technologies, such as machine learning models, to provide a theoretical basis for clinical decision-making. The study also acknowledges limitations, such as a wide temporal span of included cases and incomplete data reporting, which may affect the generalizability of findings.

## Acknowledgments

We sincerely thank all the authors for their joint efforts.

## Author contributions

**Conceptualization:** Menghui He, Xiaoqing Jin, Chunming Xian, Zhongsheng Lu, Pei Han.

**Funding acquisition:** Qiang Zhang.

**Investigation:** Xiaoqing Jin, Pei Han.

**Methodology:** Menghui He, Xiaoqing Jin, Zhongsheng Lu.

**Project administration:** Menghui He, Chunming Xian, Zhongsheng Lu, Qiang Zhang.

**Software:** Menghui He.

**Supervision:** Xiaoqing Jin, Chunming Xian, Zhongsheng Lu, Qiang Zhang, Pei Han.

**Validation:** Menghui He, Xiaoqing Jin.

**Visualization:** Menghui He, Xiaoqing Jin.

**Writing – original draft:** Menghui He.

**Writing – review & editing:** Menghui He, Zhongsheng Lu.
